# Complete Genome Sequence of Rhodococcus qingshengii Strain PM1, Isolated from a Selenium-Rich Mine in China

**DOI:** 10.1128/mra.01007-22

**Published:** 2022-12-05

**Authors:** Mu Peng, Dandan Zhang, Chao Wang, Zhihui Jiang, Xiufang Huang, Fangzhen Zhou, Fenglan Huang, Zhiyong Wang

**Affiliations:** a College of Biological Science and Technology, Hubei Minzu University, Enshi, Hubei, China; b Hubei Key Laboratory of Biological Resources Protection and Utilization, Hubei Minzu University, Enshi, Hubei, China; c Zibo Academy of Agricultural Sciences, Zibo, Shangdong, China; d College of Life Science and Food Engineering, Inner Mongolia Minzu University, Tongliao, China; Wellesley College

## Abstract

Rhodococcus qingshengii PM1 was isolated from selenium-rich carbonaceous mudstones in Enshi, Hubei, China. Here, we report the complete genome sequence of this strain, which was obtained by combining Illumina and Nanopore sequencing.

## ANNOUNCEMENT

The species Rhodococcus qingshengii was first isolated from a carbendazim-contaminated soil sample (1). Although this species is a typical soil inhabitant, it has been found in diverse habitats such as sludge, sawdust, and marine environments (2–4). Some R. qingshengii species have the capacity to promote plant growth and degrade crude oil, hazardous chemical compounds, and dyes (4–6). To date, no report about R. qingshengii isolated from selenium-contaminated sites has been published.

PM1 was isolated from selenium-rich carbonaceous mudstones in Yutangba, Enshi District, Hubei, China (1,600 m above sea level; 46°10′N, 109°46′E). A ~1-g soil sample was vortex-mixed in 50 mL sterile distilled water, and 100 μL of the slurry was plated at 28°C on Luria-Bertani (LB) agar for 3 to 5 days. The colonies were picked up and restreaked on LB agar at least five times, to ensure that the cultures were pure, and then were stored at −80°C. Strain PM1 was recovered from storage, grown at 160 rpm and 28°C in LB medium for 2 days, and harvested for genomic DNA extraction. Genomic DNA was extracted using the QIAamp DNA minikit (Qiagen, Hilden, Germany) according to the manufacturer’s protocol. For Illumina sequencing, 100 ng DNA was randomly sheared to ~300 bp by Covaris (USA), and end-repair, dA-tailing, and adapter ligation procedures were performed using the NEBNext Ultra II DNA library preparation kit for Illumina (New England Biolabs, USA). Paired-end sequencing (2 × 150 bp) was performed on the NovaSeq 6000 platform (Illumina Inc., USA). For Nanopore sequencing, the genome library was produced with no shearing using the ligation sequencing kit 1D (SQK-LSK109) with the native barcoding expansion kit (EXP-NBD104/114; Oxford Nanopore Technologies [ONT], Oxford, England). Then, the genome libraries were loaded and sequenced using a PromethION sequencer (ONT). All software was used with default parameters unless otherwise noted. Illumina raw reads (15,011,196 reads [read length, 2,251,679,400 bp]) were cleaned and trimmed using fastp v0.23.2 (7). Nanopore raw reads (373,741 reads [*N*_50_, 25,145 bp]) were quality controlled and trimmed using Guppy v5.0.16 with the high-accuracy base-calling mode (implemented in the MinKNOW interface). *De novo* assembly and circularization were performed in a hybrid genome assembly and the assembly was rotated to start with *dnaA* using Unicycler v0.5.0 (8). The ONT contigs were polished with the Illumina short reads using Pilon v1.22 (9). Genome annotation was performed using the NCBI Prokaryotic Genome Annotation Pipeline (PGAP) v6.3 with the best-placed reference protein set and GeneMarkS-21 (10).

PM1 was identified as R. qingshengii based on its average nucleotide identity (ANI) of 98.46% (11) and its 16S rRNA gene nucleotide identity of 100% with respect to R. qingshengii JCM 15477 (GenBank accession number CP096563.1). The genome size of PM1 is 6,373,845 bp, with a GC content of 62.84% and high average coverage levels (326× for Illumina sequencing and 721× for Nanopore sequencing). A total of 5,893 genes were annotated, including 5,802 coding genes, 70 RNA genes, and 21 pseudogenes.

### Data availability.

The complete genome sequence has been deposited in GenBank under accession number CP104782, BioProject accession number PRJNA881770, and SRA accession numbers SRR21679470 (Illumina sequencing) and SRR21679469 (Nanopore sequencing).
